# Topical glycopyrronium tosylate in Japanese patients with primary axillary hyperhidrosis: A randomized, double‐blind, vehicle‐controlled study

**DOI:** 10.1111/1346-8138.16188

**Published:** 2021-10-11

**Authors:** Hiroo Yokozeki, Tomoko Fujimoto, Shunsuke Wanatabe, Shuhei Ogawa, Chie Fujii

**Affiliations:** ^1^ Department of Dermatology Graduate School of Medical and Dental Sciences Tokyo Medical and Dental University Tokyo Japan; ^2^ Ikebukuro Nishiguchi Fukurou Dermatology Clinic Tokyo Japan; ^3^ Clinical Development Department Maruho Co., Ltd. Kyoto Japan

**Keywords:** antiperspirants, axilla, cholinergic antagonists, glycopyrronium tosylate, hyperhidrosis

## Abstract

Glycopyrronium tosylate cloth, an anticholinergic drug, has been approved for the topical treatment of primary axillary hyperhidrosis in the USA, but its effects in Japanese patients have not been previously investigated. This 4‐week, randomized, double‐blind, vehicle‐controlled, multicenter study was conducted to evaluate the efficacy and safety of glycopyrronium tosylate cloth for primary axillary hyperhidrosis patients in Japan. Eligible patients, who were ≥9 years of age and had primary axillary hyperhidrosis ≥6 months, with gravimetrically‐measured sweat production ≥50 mg/5 min, and Hyperhidrosis Disease Severity Scale ≥3 (moderate) were randomized 1:1:1 to once daily topical glycopyrronium tosylate 3.75%, 2.5%, or vehicle. Overall, 497 patients (163 in the glycopyrronium tosylate 3.75% group, 168 in the glycopyrronium tosylate 2.5% group, and 166 in the vehicle group, hereinafter in this order) were randomized. Statistically higher proportions of patients in the glycopyrronium tosylate groups achieved ≥2‐point improvement in Hyperhidrosis Disease Severity Scale and ≥50% reduction in sweat production from baseline versus vehicle at week 4 (51.6%, 41.1%, and 16.4%, respectively; *p* < 0.001 in both cases). Higher responder rates in the glycopyrronium tosylate groups compared with the vehicle group occurred as early as week 1. The most common treatment‐emergent adverse events in patients treated with glycopyrronium tosylate were photophobia, mydriasis, thirst, and dysuria. Most treatment‐emergent adverse events were mild as determined by the investigators. The incidence of treatment‐emergent adverse events leading to treatment modification was low in the three groups. The 4‐week use of topical glycopyrronium tosylate improved the patient‐reported outcome measure Hyperhidrosis Disease Severity Scale and objectively‐evaluated sweat production with a favorable benefit/risk profile.

## 1 INTRODUCTION

Hyperhidrosis is a skin disorder characterized by excess sweating at a level more than required to maintain normal body temperature. Primary hyperhidrosis is idiopathic, caused by overactive sympathetic nerves, and involves a limited body area, most often the axillae, palms, soles, or craniofacial region.^1^ Primary axillary hyperhidrosis affects approximately 5.75% of Japanese children and adults,^2^ which is much higher than the prevalence in the USA (1.4%).^3^ Patients with primary axillary hyperhidrosis usually experience excess underarm sweating, and the disease presents bilaterally with often other body regions also affected.^1^ The condition also negatively impacts patient quality of life (QOL) by reducing work productivity and affecting mental health.^1^, ^3^, ^4^, ^5^, ^6^, ^7^, ^8^ Despite its high prevalence, significant symptoms, and impact on QOL, most sufferers do not seek medical treatment or wait for a few years before seeking medical help,^9^ likely because of the insufficient recognition of primary axillary hyperhidrosis as an intractable and potentially disabling disease with limited treatment options available.^10^ In a recent study, the clinical efficacy and safety of a topical cholinergic antagonist, sofpironium bromide, were demonstrated.^11^

Glycopyrronium tosylate (GT) is a competitive inhibitor of acetylcholine receptors, which are expressed in various peripheral tissues, including the sweat glands. GT 3.75% was approved by the US Food and Drug Administration (FDA) in 2018 as a topical anticholinergic drug for primary axillary hyperhidrosis in patients ≥9 years of age.^6^, ^12^, ^13^, ^14^, ^15^, ^16^, ^17^, ^18^ However, clinical data for the use of GT in Japanese patients have not been reported.

We developed two doses (2.5% and 3.75%) of a new GT formulation (cloth) with a different ethanol concentration for use in Japan. In this phase II/III study, the efficacy and safety of GT cloth were evaluated in Japanese patients with primary axillary hyperhidrosis.

## 2 METHODS

### 2.1 Study design

This study was approved by the institutional review board at each study site (Appendix S1) and conducted in accordance with the principles of the Declaration of Helsinki, Good Clinical Practice, and other applicable regulations. All patients and/or their legal guardians provided written informed consent prior to participating in this study. The trial registration number was JapicCTI‐194819, accorded to the Clinical Development Department, Maruho, for the Topical Glycopyrronium Tosylate Study Group.

This phase II/III, 4‐week randomized, double‐blind, parallel‐group, vehicle‐controlled, multicenter study was conducted from July 2019 to December 2019 at 49 study sites in Japan. A list of principal investigators is shown in Appendix S2. After a 7‐day screening period, eligible patients with primary axillary hyperhidrosis were randomized 1:1:1 to once daily GT 3.75% (equivalent to 2.4% glycopyrronium), GT 2.5% (equivalent to 1.6% glycopyrronium), or a matched vehicle group using an interactive web‐based response system (day 1). Investigators, study site staff, patients, and the sponsor were all blinded to treatment assignment throughout the study. Patients were asked to apply GT 3.75%, GT 2.5%, or vehicle to the cleaned and dried skin of both axillae before bedtime, and to wash their hands after each application and to not wash the axillae for at least 4 h after application. Patients could shave hair in each axilla on the morning of each dosing day.

During the study, the following medications and treatments for axillae and hyperhidrosis were prohibited: antiperspirants, glycopyrronium agents, cholinergics, anticholinergics, clonidine, Chinese herbal drugs, iontophoresis, botulinum toxin, surgery or psychotherapy, and actions that might damage the axilla.

### 2.2 Patients

Patients included in this study were ≥9 years of age who had primary axillary hyperhidrosis for at least 6 months, had sweat production of at least 50 mg/5 min for each axilla, and had a Hyperhidrosis Disease Severity Scale (HDSS) of 3 (moderate) or 4 (severe). Patients with any of the following conditions were excluded from the study: hypersensitivity to alcohol that required drug treatment or glycopyrronium‐containing products; history of surgical procedure for hyperhidrosis; prior axillary treatment with topical agents within 2 weeks of screening; prior axillary treatment with iontophoresis within 4 weeks of screening; treatment with botulinum toxin within 1 year of screening; or other treatments with cholinergic or anticholinergic activity within 4 weeks of screening. A full list of inclusion and exclusion criteria is presented in Appendix S3.

### 2.3 Efficacy and safety assessments

#### 2.3.1 Efficacy endpoints

The primary efficacy endpoint was the proportion of patients achieving a HDSS ≥2‐point improvement (HDSS responder rate) and a ≥50% reduction in sweat production (SP) (mean of both axillae) (50% SP responder rate) from baseline at week 4 (HDSS responder and 50% SP responder rate). Patients acclimatized to the controlled room conditions at each study site for at least 30 min before sweat was collected. Sweat production was measured in a temperature (20–28°C) and humidity‐controlled room with the gravimetric sweat method, which uses a pre‐weighed filter paper applied to the affected axilla area for 5 min, and sweat production was assessed as the difference in weight of the filter paper by subtracting the post‐application filter weight from the pre‐weighed filter weight. The assessment of sweat production was carried out with the same timing where possible for each patient. The HDSS is a hyperhidrosis‐specific patient‐reported outcome measure using a 4‐point scale with higher scores indicating a greater negative effect on daily life, which has been validated and used in numerous studies.^19^

Secondary efficacy endpoints were the HDSS responder rate at week 4, the 50% SP responder rate at week 4, and the proportion of patients achieving Axillary Sweating Daily Diary (ASDD)/ASDD for Children (ASDD‐C) Item 2 with a ≥4‐point improvement (ASDD/ASDD‐C responder rate) at week 4, and sweat production at week 4. The ASDD, a FDA‐validated patient‐reported outcome measure, comprises six items to assess the severity, impact, and bothersomeness of axillary hyperhidrosis experienced by patients. Patients ≥9 to <16 years of age used the ASDD‐C (comprising the first two items of ASDD) to measure the severity of sweating. For the ASDD and ASDD‐C Item 2 scales that are 11‐point scales (0–10) similar to the HDSS, higher scores indicated more severe sweating.^20^, ^21^ ASDD/ASDD‐C scores were recorded in an electronic diary provided by the sponsor.

#### 2.3.2 Exploratory endpoints

Weekly trends in the HDSS responder rate, 50% or 75% SP responder rate, sweat production, ASDD/ASDD‐C responder rate, Dermatology Life Quality Index (DLQI)/Children’s DLQI (CDLQI) scores, HDSS, and ASDD/ASDD‐C Item 2 scores were also evaluated from baseline to week 4, as exploratory endpoints. The 0–30 scale DLQI (patients ≥16 years of age)/CDLQI (patients 9–15 years of age) was used to assess the impact of dermatology‐related diseases on patient health‐related QOL, with higher scales indicating lower QOL.^22^, ^23^

#### 2.3.3 Safety endpoints

Treatment‐emergent adverse events (TEAE), clinical laboratory tests, and vital signs were assessed in the safety evaluation throughout the 4‐week treatment period. TEAE of special interest were defined as symptoms associated with mydriasis/blurry vision and dysuria/urinary retention.

### 2.4 Statistical analysis

Randomization of 150 patients in each treatment group was estimated to provide >90% power at a significance level of 0.05 to detect a difference when comparing the primary efficacy endpoint between GT 3.75% and vehicle and GT 2.5% and vehicle, assuming a vehicle response rate of 20% and a 20% risk difference between GT 3.75% and vehicle and GT 2.5% and vehicle based on previous studies.^17^

Efficacy analyses were conducted in the modified intention‐to‐treat (mITT) population, which included all patients who were randomized and treated with GT or vehicle.

Primary efficacy endpoint testing among doses was adjusted for multiplicity using a closed testing procedure. A comparison between GT 2.5% and vehicle was tested only when the statistical significance between GT 3.75% and vehicle was confirmed at a two‐sided alpha level of 0.05. No adjustment was made for multiplicity for the secondary endpoints. The primary efficacy endpoint and other binary secondary endpoints were analyzed using Pearson’s χ^2^‐test. Log‐transformed gravimetrically‐measured sweat production was calculated for secondary and exploratory endpoints, and conditional longitudinal data analysis was used to compare sweat production between groups. Missing data were imputed as a non‐responder for binary endpoints but were not imputed for continuous endpoints. One pre‐specified sensitivity analysis was performed for the primary efficacy endpoint: Pearson’s χ^2^‐test with last observation carried forward imputation for missing data. All statistical analyses were conducted using SAS version 9.4 software (SAS Institute).

## 3 RESULTS

### 3.1 Patient disposition, demographics, and baseline characteristics

**FIGURE 1:**
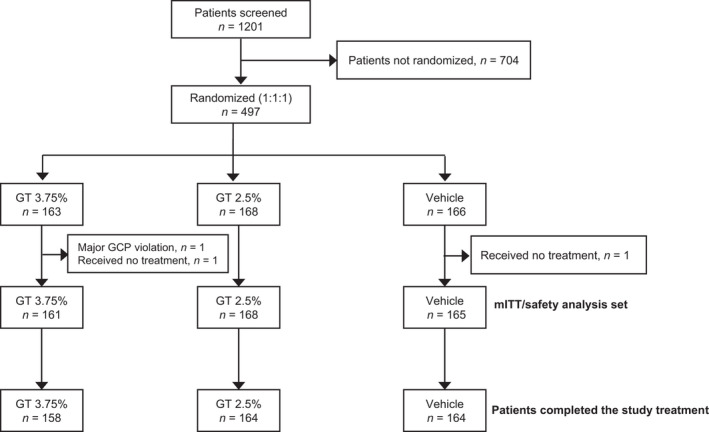
Disposition of patients. GCP, Good Clinical Practice; GT, glycopyrronium tosylate; mITT, modified intention‐to‐treat.

Overall, 497 patients were randomly assigned to the GT 3.75% (163 patients), GT 2.5% (168 patients), or vehicle (166 patients) group (hereinafter in this order) (Figure 1). Two patients in the GT 3.75% group (one each for Good Clinical Practice violation and not treated) and one patient in the vehicle group (not treated) were excluded from the analyses. Therefore, 161, 168, and 165 patients were included in the mITT.

Of patients included in the mITT, 97.8% (158, 164, and 164) of patients completed the 4‐week treatment. Baseline characteristics of patients in the three groups were similar (Table 1). In all groups, over 75% of patients had HDSS 3, with a median sweat production >100 mg/5 min. Approximately 87% of patients had no treatment history for primary axillary hyperhidrosis, with a mean ASDD/ASDD‐C Item 2 of approximately 6.0, and approximately 30% of patients also had palmar, plantar, face, scalp, and/or trunk hyperhidrosis.

**TABLE 1:** Baseline demographic and clinical characteristics (modified intention‐to‐treat population)

Parameter	3.75% GT (n = 161)	2.5% GT (n = 168)	Vehicle (n = 165)
Sex, female, no. (%)	100 (62.1)	97 (57.7)	94 (57.0)
Age, years (mean, SD)	38.6 (12.2)	38.3 (10.8)	37.7 (10.9)
<16 years	0	0	2 (1.2)
≥16 years	161 (100)	168 (100)	163 (98.8)
Bodyweight, kg (mean, SD)	59.9 (11.2)	61.6 (13.0)	61.3 (12.1)
Age of onset, years (mean, SD)	n = 152	n = 157	n = 156
	18.4 (8.8)	18.5 (8.2)	19.0 (8.0)
Duration of primary axillary hyperhidrosis, years (mean, SD)	n = 152	n = 157	n = 156
	21.1 (12.3)	20.2 (11.7)	19.1 (9.7)
Family history^a^, no. (%)			
None	81 (50.3)	84 (50.0)	96 (58.2)
Sibling	20 (12.4)	20 (11.9)	21 (12.7)
Parent	51 (31.7)	62 (36.9)	43 (26.1)
Child	16 (9.9)	14 (8.3)	11 (6.7)
Grandparent	4 (2.5)	1 (0.6)	1 (0.6)
Grandchild	0	0	0
Treatment history for axillary hyperhidrosis^a^, no. (%)			
None	140 (87.0)	146 (86.9)	148 (89.7)
Yes	21 (13.0)	22 (13.1)	17 (10.3)
Aluminum chloride topical	5 (3.1)	8 (4.8)	5 (3.0)
Topical anticholinergics	6 (3.7)	3 (1.8)	4 (2.4)
Oral anticholinergics	1 (0.6)	1 (0.6)	0
Other	10 (6.2)	11 (6.5)	8 (4.8)
Body region impacted^a^, no. (%)			
Palmar	62 (38.5)	59 (35.1)	62 (37.6)
Plantar	53 (32.9)	51 (30.4)	50 (30.3)
Face	57 (35.4)	53 (31.5)	49 (29.7)
Scalp	48 (29.8)	38 (22.6)	42 (25.5)
Trunk	58 (36.0)	65 (38.7)	55 (33.3)
HDSS for primary axillary hyperhidrosis			
Grade 3	122 (75.8)	128 (76.2)	126 (76.4)
Grade 4	39 (24.2)	40 (23.8)	39 (23.6)
Sweat production (mg/5 min), median (Q1–Q3)	106.7 (73.6, 166.3)	102.5 (74.8, 174.9)	108.4 (69.1, 153.7)
ASDD/ASDD‐C Item 2 score^b^, (mean, SD)	6.0 (1.7)	6.1 (1.6)	5.9 (1.7)
DLQI^c^ (mean, SD)	7.9 (5.7)	7.0 (5.5)	6.7 (5.2)
CDLQI^d^ (mean, SD)	–	–	1.5 (2.1)

Note

All patients included in each group in the trial were Asian (100%).

Abbreviations: ASDD, Axillary Sweating Daily Diary; ASDD‐C, ASDD for Children; CDLQI, Children’s Dermatology Life Quality Index; DLQI, Dermatology Life Quality Index; GT, glycopyrronium tosylate; HDSS, Hyperhidrosis Disease Severity Scale; SD, standard deviation.

^a^ Patients could report more than one response; therefore, percentages may total more than 100%.

^b^ Patients ≥16 years of age were assessed using ASDD and those <16 years of age were assessed using ASDD‐C.

^c^ Assessed in patients ≥16 years of age.

^d^ Assessed in patients <16 years of age.

### 3.2 Efficacy

**FIGURE 2:**
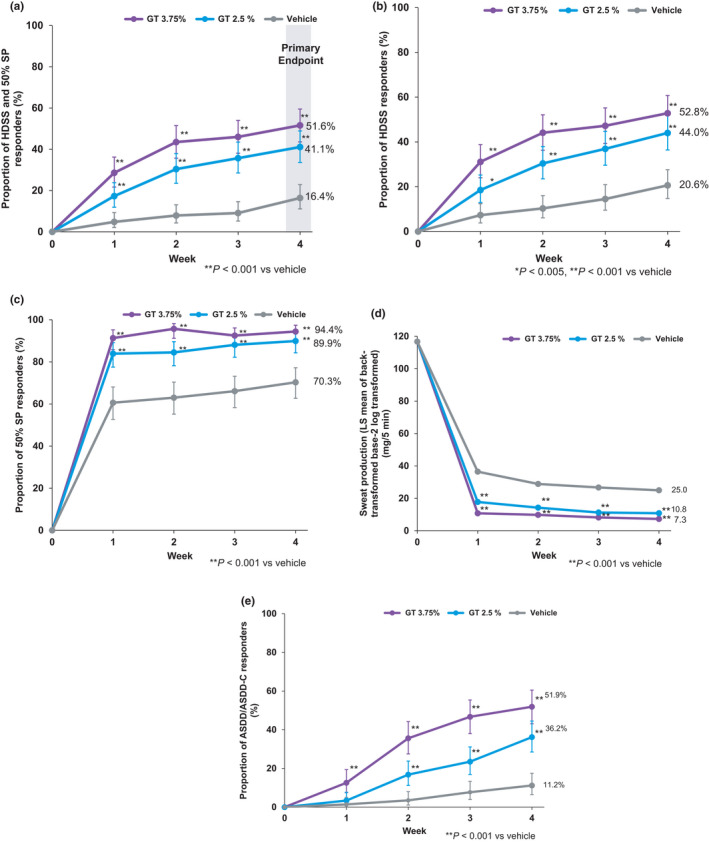
Efficacy of topical glycopyrronium tosylate cloth (GT 3.75% and GT 2.5%) in patients with primary axillary hyperhidrosis. (a) Proportion of HDSS responders and 50% SP responders, (b) proportion of HDSS responders, (c) proportion of 50% SP responders, (d) sweat production, and (e) proportion of ASDD/ASDD‐C responders. (a–c,e) Values are the % (exact 95% CI). (d) Values are the LS mean. Patients ≥16 years of age were assessed using ASDD Item 2 and those <16 years of age were assessed using ASDD‐C Item 2. Missing data were imputed as a non‐response for binary endpoints but were not imputed for the continuous endpoints. ASDD, Axillary Sweating Daily Diary; ASDD‐C, ASDD for Children; CI, confidence interval; GT, glycopyrronium tosylate; HDSS, Hyperhidrosis Disease Severity Scale; LS mean, least squares mean; SP, sweat production. [Color figure can be viewed at wileyonlinelibrary.com]

The primary endpoint, HDSS responder, and 50% SP responder rate at week 4 was 51.6% (83/161 patients) in the GT 3.75% group (*p* < 0.001) and 41.1% (69/168) in the GT 2.5% group (*p* < 0.001) versus 16.4% (27/165) in the vehicle group (Table 2). Higher responder rates in GT groups compared with the vehicle group occurred as early as week 1 for both GT concentrations (Figure 2a). A greater improvement was observed for GT 3.75% than for GT 2.5%. A similar result was obtained for the primary endpoint in the sensitivity analysis (data not shown).

**TABLE 2:** Efficacy endpoints at week 4 (modified intention‐to‐treat population)

Parameter	3.75% GT (n = 161)	2.5% GT (n = 168)	Vehicle (n = 165)
Primary endpoint			
HDSS and SP responder rate^a^, no. (%)	83 (51.6)	69 (41.1)	27 (16.4)
Difference vs. vehicle (95% CI^h^)	35.2 (24.8–45.1) *p* < 0.001	24.7 (14.0–34.8) *p* < 0.001	–
Secondary endpoint			
HDSS responder rate^b^, no. (%)	85 (52.8)	74 (44.0)	34 (20.6)
Difference vs. vehicle (95% CI^h^)	32.2 (21.6–42.2) *p* < 0.001	23.4 (12.8–33.7) *p* < 0.001	–
SP responder rate^c^, no. (%)	152 (94.4)	151 (89.9)	116 (70.3)
Difference vs. vehicle (95% CI^h^)	24.1 (13.5–34.5) *p* < 0.001	19.6 (8.8–30.1) *p* < 0.001	–
Mixed model for sweat production			
Base‐2 log‐transformed, LS mean (SE)	2.8656 (0.1391)	3.4361 (0.1363)	4.6420 (0.1369)
Difference vs. vehicle (95% CI)	−1.7765 (−2.1569 to −1.3960)	−1.2059 (−1.5824 to −0.8295)	–
Original scale, LS mean (mg/5 min)^d^	7.2884 *p* < 0.001	10.8236 *p* < 0.001	24.9687

	n = 135	n = 149	n = 143
ASDD/ASDD‐C^e^ Item2 responder rate^f^, no. (%)	70 (51.9)	54 (36.2)	16 (11.2)
Difference vs. vehicle (95% CI^h^)	40.7 (29.4–51.0) *p* < 0.001	25.1 (13.6–35.9) *p* < 0.001	–

Exploratory efficacy endpoint	n = 161	n = 168	n = 162
Change in DLQI^g^ score from baseline, mean (SD)	−6.5 (5.6)	−4.8 (5.1)	−3.6 (4.9)
Difference vs. vehicle (95% CI)	−2.9 (−4.0 to −1.7) *p* < 0.001	−1.2 (−2.2 to −0.1) *p* = 0.036	
Proportion of patients with a ≥75% reduction in sweat production from baseline, no. (%)	135 (83.9)	128 (76.2)	85 (51.5)
Difference vs. vehicle (95% CI^h^)	32.3 (21.6–42.2) *p* < 0.001	24.7 (14.0–34.8) *p* < 0.001	–

Abbreviations: ASDD, Axillary Sweating Daily Diary; ASDD‐C, ASDD for Children; CI, confidence interval; DLQI, Dermatology Life Quality Index; GT, glycopyrronium tosylate; HDSS, Hyperhidrosis Disease Severity Scale; LS mean, least squares mean; SD, standard deviation; SE, standard error; SP, sweat production.

^a^ Proportion of patients achieving an HDSS ≥2‐point improvement and a ≥50% reduction in sweat production from baseline.

^b^ Proportion of patients with a ≥2‐point improvement in HDSS from baseline.

^c^ Proportion of patients with a ≥50% reduction in sweat production from baseline.

^d^ Back‐transformed value.

^e^ Patients ≥16 years of age were assessed using ASDD and those <16 years of age were assessed using ASDD‐C.

^f^ Proportion of patients with a ≥4‐point improvement in weekly mean score of ASDD/ASDD‐C Item 2 from baseline.

^g^ Assessed in patients ≥16 years of age. The primary efficacy endpoint and other binary secondary endpoints were analyzed with Pearson’s χ^2^‐test. Conditional longitudinal data analysis was used to compare sweat production. Primary efficacy endpoint testing among doses was adjusted for multiplicity using a closed testing procedure. Secondary endpoints were not adjusted for multiplicity. Missing data were imputed as non‐response for binary endpoints but were not imputed for continuous endpoints.

^h^ Exact confidence interval.

As summarized in Table 2, the secondary endpoints (HDSS responder rate, 50% SP responder rate, and ASDD/ASDD‐C responder rate) and sweat production at week 4 showed greater improvements for GT 3.75% and GT 2.5% versus vehicle (*p* < 0.001 for both GT groups vs. vehicle group for all three secondary endpoints, without multiplicity adjustment). Time‐dependent improvements in the GT groups were also observed as early as week 1 for the HDSS responder rate (Figure 2b), 50% SP responder rate (Figure 2c), sweat production (Figure 2d), and ASDD/ASDD‐C responder rate (Figure 2e). In addition, a significantly greater proportion of GT 3.75%‐ and GT 2.5%‐treated patients achieved the 75% SP responder rate at week 4 (*p* < 0.001 for both GT groups), and GT groups also had a significantly greater mean decrease (improvement) in DLQI versus the vehicle group (GT 3.75%, *p* < 0.001; GT 2.5%, *p* < 0.05) (Table 2).

### 3.3 Safety

A similar proportion of patients experienced TEAE in the GT 3.75%, GT 2.5%, and vehicle groups (36.0%, 30.4%, and 33.9%, respectively) (Table 3). One patient in the vehicle group experienced a serious TEAE. Most TEAE were mild as determined by the investigators, except for those in two patients: one patient in the vehicle group experienced severe putamen hemorrhage, and another patient in the GT 2.5% group experienced a moderate treatment‐related urine output decrease, which was recovered, although the patient continued the study treatment without any modification even after the onset of urine output decreased.

**TABLE 3:** Treatment‐emergent adverse events (safety population)

TEAE^a^	3.75% GT (n = 161)	2.5% GT (n = 168)	Vehicle (n = 165)
TEAE, no. of patients (%)			
All	58 (36.0)	51 (30.4)	56 (33.9)
Related to study treatment	39 (24.2)	26 (15.5)	22 (13.3)
Death	0	0	0
Serious adverse event	0	0	1 (0.6)^b^
Treatment modification			
Discontinuation	3 (1.9)	0	1 (0.6)
Interruption	3 (1.9)	2 (1.2)	0
Reduction	0	0	0
TEAE by intensity			
Mild	58 (36.0)	50 (29.8)	56 (33.9)
Moderate	0	1 (0.6)	0
Severe	0	0	1 (0.6)
TEAE of special interest			
Mydriasis/blurred vision^c^	18 (11.2)	13 (7.7)	6 (3.6)
Dysuria/urinary retention^d^	14 (8.7)	8 (4.8)	9 (5.5)
TEAE reported by >2% of patients in either group			
Mydriasis	6 (3.7)	6 (3.6)	1 (0.6)
Photophobia	10 (6.2)	4 (2.4)	1 (0.6)
Blurred vision	2 (1.2)	3 (1.8)	4 (2.4)
Thirst	8 (5.0)	3 (1.8)	5 (3.0)
Nasopharyngitis	11 (6.8)	11 (6.5)	13 (7.9)
Dysuria	6 (3.7)	5 (3.0)	3 (1.8)
Pollakiuria	5 (3.1)	2 (1.2)	5 (3.0)
Oropharyngeal pain	5 (3.1)	0	0
TEAE occurring at application site	5 (3.1)	3 (1.8)	3 (1.8)
Application site dermatitis	1 (0.6)	2 (1.2)	0
Application site irritation	0	1 (0.6)	0
Application site pruritus	0	0	1 (0.6)
Folliculitis	1 (0.6)	0	0
Application site folliculitis	0	0	2 (1.2)
Wound	1 (0.6)	0	0
Acne	1 (0.6)	0	0
Eczema asteatotic	1 (0.6)	0	0

Note

Data reported as n (%).

Abbreviations: GT, glycopyrronium tosylate; MedDRA, Medical Dictionary for Regulatory Activities; TEAE, treatment‐emergent adverse event.

^a^ Classified using MedDRA/J version 22.0.

^b^ Putamen hemorrhage.

^c^ Mydriasis, pupils unequal, hypermetropia, blurred vision, and photophobia.

^d^ Urinary hesitation, urinary retention, urine flow decreased, pollakiuria, dysuria, nocturia, and urine output decreased.

Few TEAE led to the discontinuation of study treatment (three patients and one patient in the GT 3.75% and vehicle groups, respectively). These included dysuria and thirst in three patients each, constipation in two patients, and dry eye and oropharyngeal pain in one patient each in the GT 3.75% group. All of these were treatment‐related and all were recovered (Table S1). The incidence of TEAE leading to an interruption of study treatment was also low (three patients and two patients in the GT 3.75% and 2.5% groups, respectively). The TEAE included dysuria, mydriasis, photophobia, and pollakiuria (all in one patient each) in the GT 3.75% group, and mydriasis and dry mouth in one patient each in the GT 2.5% group, which were all recovered or recovering.

Regarding TEAE of special interest, the incidence of mydriasis/blurred vision was 11.2% in the GT 3.75% group, 7.7% in the GT 2.5% group, and 3.6% in the vehicle group, and the incidence of dysuria/urinary retention was 8.7%, 4.8%, and 5.5%, respectively. All TEAE of special interest that occurred in the GT groups were treatment‐related.

The incidences of TEAE (potentially attributed to the anticholinergic effects of GT), including mydriasis, photophobia, and dysuria in the GT groups were higher than those in the vehicle group. TEAE that occurred in more patients in the GT groups than the vehicle group included mydriasis (3.7%, 3.6%, and 0.6%), photophobia (6.2%, 2.4%, and 0.6%), dysuria (3.7%, 3.0%, and 1.8%), and oropharyngeal pain (3.1%, 0.0%, and 0.0%). Overall, the incidences of TEAE that occurred at the application site were low (3.1% in the GT 3.75% group, and 1.8% in the GT 2.5% and vehicle groups). No clinically meaningful changes in the results of the laboratory tests or vital signs were observed.

## 4 DISCUSSION

The current study demonstrated that a once‐daily 4‐week topical treatment with GT 3.75% or 2.5% cloth improved the HDSS and sweat production in patients with primary axillary hyperhidrosis. These results were further supported by the findings related to the secondary and exploratory endpoints.

The primary endpoint of this study was the proportion of patients who achieved HDSS with a ≥2‐point improvement and a ≥50% reduction in sweat production, which was designed to enable the subjective and objective evaluation of the efficacy of GT. Notably, the effects of GT became apparent at week 1, which indicated the rapid action of GT. These results demonstrate the clinically meaningful and statistically significant improvement of GT treatment. The efficacies of GT assessed using the HDSS and sweat production are similar to those previously reported outside Japan.^13^, ^15^, ^17^

In this study, a composite endpoint comprising the HDSS and sweat production was used as the primary endpoint. Our results demonstrate that most patients who achieved a HDSS of 1 or 2 with a ≥2‐point improvement also achieved a ≥50% reduction in sweat production. Indeed, the data shown in Figure 2(a,b) are very similar. This suggests that the HDSS responder rate and 50% SP responder rate were similar to the HDSS responder rate. Based on these results, the HDSS responder rate might be used as the only primary endpoint in future studies to evaluate clinically meaningful improvements in patients with hyperhidrosis by GT. It should be noted that a high vehicle effect on sweat production was observed in this study, as previously reported in patients with primary axillary hyperhidrosis.^11^, ^13^

The efficacy of GT on patient QOL was evaluated using the DLQI in patients ≥16 years of age. Overall, changes in the DLQI scores were significantly decreased (improved) from baseline at week 4 in the GT 3.75% group (6.5) and 2.5% group (4.8). The mean reductions in the DLQI in both GT groups indicated a clinically meaningful improvement based on a previous study that described a minimum clinically important difference of 2.8–4.6.^17^

Axillary Sweating Daily Diary and ASDD‐C are axillary hyperhidrosis‐specific patient‐reported outcome measures that evaluate the severity, impact, and bothersomeness of axillary hyperhidrosis,^20^, ^21^ but only their English‐language versions have been validated. Because it is a 0–10 numerical rating scale (commonly used in dermatology clinical trials), the ASDD Item 2 could be used for Japanese and English natives in the trial. In the current study, the results with ASDD Item 2 were consistent with those of previous studies suggesting ASDD Item 2 might be useful to assess the severity of axillary hyperhidrosis and primary hyperhidrosis other than axillae in Japanese patients.

Overall, daily GT treatment was well‐tolerated in patients in both GT groups. All TEAE in the GT groups were mild, except for moderate urine output decrease in one patient in the GT 2.5% group. Regarding TEAE of special interest, higher incidences of treatment‐related TEAE were found in the GT 3.75% group compared with the GT 2.5% and vehicle groups. These included mydriasis/blurred vision in 11.2% of patients in the GT 3.75% group and 7.7% of patients in the GT 2.5% group that occurred in one or both eyes. In addition to the systemic anticholinergic effects, GT adhered to the hands might have caused local anticholinergic effects, resulting in the events of mydriasis/blurred vision. Therefore, it is important to instruct patients to wash their hands adequately after each application. Furthermore, the incidence of TEAE that occurred at the application site was low, which suggests that GT 3.75% and GT 2.5% are unlikely to cause clinically significant irritation. Of note, the results of the present study showed a favorable benefit/risk profile compared with those previously reported.^13^ Therefore, fast‐acting, effective, and well‐tolerated treatment with GT is expected to reduce the physical and psychological burdens in patients with primary hyperhidrosis.

This study had some limitations. This study evaluated the efficacy and safety of GT for 4 weeks. Because patients will need to be treated with GT for a longer duration, the long‐term efficacy and safety of GT are currently under investigation. Another limitation is that although a previous Japanese epidemiological study reported that the prevalence of primary focal hyperhidrosis was 4.27% for patients aged between 5 and 9 years,^2^ no patient younger than 14 years of age was enrolled in the present study despite a lower age limit of 9 years. Finally, we targeted primary axillary hyperhidrosis in this study. However, patients with axillary hyperhidrosis often have other affected areas,^2^ as observed in the current study. To improve the overall primary hyperhidrosis patient QOL further, future studies could evaluate the efficacy and safety of GT for primary hyperhidrosis at other affected body areas.

In conclusion, the 4‐week use of topical GT 3.75% and GT 2.5% cloths for primary axillary hyperhidrosis resulted in a clinically meaningful and statistically significant reduction in the severity of hyperhidrosis and sweat production compared with the vehicle, and the daily application of GT was generally well tolerated. GT cloth is a non‐invasive, non‐dispensing, and self‐administrated topical agent. GT cloth is highly convenient for patients and is expected to be widely prescribed by physicians compared with topical aluminum chloride or botulinum toxin type A injection as recommended in the Clinical Guidelines for primary axillary hyperhidrosis.^10^ Therefore, daily treatment with GT cloth might be a first‐line treatment for patients with primary axillary hyperhidrosis in Japan.

## CONFLICT OF INTEREST

H.Y. and T.F. received research funding, consultancy fees, speaker fees, fees for arranging education, and personal fees from Maruho. S.W., S.O., and C.F., are employees of Maruho.

## Supporting information

Supplementary Material
