# Supplementary material for: Topical glycopyrronium tosylate in Japanese patients with primary axillary hyperhidrosis: A randomized, double‐blind, vehicle‐controlled study
Source: J Dermatol. 2021 Oct 11;49(1):86–94. doi: 10.1111/1346-8138.16188 (PMC9293301; doi:10.1111/1346-8138.16188)
Supplement: Supplementary file 1 — Supplementary Material [file JDE-49-86-s001.docx]

**Supporting Information**

**Appendix S1** List of institutional review boards (IRBs)

Aichi Medical University Hospital IRB

Medical Corporation Shintokai Yokohama Minoru Clinic IRB

Maebashi Hirosegawa Clinic IRB

Nagasaki University Hospital IRB

**Appendix S2** List of principal investigators

Topical glycopyrronium tosylate Japanese study group

| Institution/site name | Name |
| --- | --- |
| Igarashi Dermatology Clinic | Masaru Igarashi |
| Kaminoge Dermatology Clinic | Akiko Ishikoh |
| Kitahara Dermatology Clinic | Hiroto Kitahara |
| Tampopo Dermatology Clinic | Yumiko Kudo |
| Tsunoda Clinic | Meiko Tsunoda |
| Akihabara Skin Clinic | Yuki Horiuchi |
| Mildix Skin Clinic | Yoshiyuki Murakami |
| Maruyama Dermatology Clinic | Ryuji Maruyama |
| Naoko Dermatology Clinic | Naoko Hattori |
| Mita Dermatology Clinic | Hiroki Kanda |
| Ikebukuro Nishiguchi Fukurou Dermatology Clinic | Tomoko Fujimoto |
| Nemunoki Dermatology | Ichiro Nakasu |
| Aichi Medical University Hospital | Yuichiro Ohshima |
| Senri-Chuo Hanafusa Dermatology Clinic | Takaaki Hanafusa |
| Matsuo Clinic | Keizo Matsuo |
| Tomoko Matsuda dermatological Clinic | Tomoko Matsuda |
| Ekihigashi Hihuka Allergy Clinic | Yuriko Egami |
| Chiharu Dermatology Clinic | Chiharu Watanabe |
| Midori Skincare Clinic | Yoshihiro Sato |
| Akemi Hifuka | Naoko Motohashi |
| Itoh Skin Clinic | Yusei Itoh |
| Matsuyama Dermatology Clinic | Tomohiko Matsuyama |
| Kato Dermatology Plastic surgery | Takeo Kato |
| Okuda Dermatology Clinic | Tomoki Okuda |
| Hamaguchi Skin Clinic | Taizo Hamaguchi |
| MIU Skin Clinic | Nobuaki Morishita |
| Tachikawa Dermatology Clinic | Hideki Ito |
| Mizutani Dermatology Clinic | Haruko Mizutani |
| Sumire Dermatology Clinic | Nobuhiro Fujita |
| Forest Palace Dermatology Clinic | Jun Ogaki |
| Sawada Dermatology Clinic | Shunichi Sawada |
| Fukuro Dermatology Clinic | Shuhei Fukuro |
| Asai Dermatology Clinic | Toshiya Asai |
| Kosugi Dermatology Clinic | Aisaku Yamamoto |
| Miyamae Ito Clinic | Masaru Ito |
| Miyamae Ito Clinic | Yuko Tsutsumi |
| Sion Hifuka Duo | Masahiro Hiramatsu |
| Dermatology and Ophthalmology Kume Clinic | Akihiro Kume |
| Yoshikawa Hifuka | Takeshi Yoshikawa |
| Okawa Dermatology Clinic | Takeshi Okawa |
| Hamaguchi Clinic | Masaaki Yamamoto |
| Hamaguchi Clinic Tennouji | Masamitsu Hamaguchi |
| Nagasaki University Hospital | Hiroyuki Murota |
| Clinique Dermatologique Todoroki | Yoko Todoroki |
| Iidabashi Skin Clinic | Shiomi Kawano |
| Sugisawa Dermatology Clinic | Yutaka Sugizawa |
| Aozora Dermatology Clinic | Masato Koda |
| Adachi Dermatological Clinic | Jun Adachi |
| Mochida Dermatology Clinic | Kazunobu Mochida |
| Moa Clinic | Masayo Nakamura |

**Appendix S3** Study inclusion and exclusion criteria

Inclusion Criteria

(1) Visit 1 (Enrollment)

1) Patients who had excessive local axillary perspiration for at least 6 months without apparent secondary causes

2) Patients must have met two or more of criteria a) - f), below, to be eligible for study participation.

a) Age of onset under 25 years

b) Bilateral and symmetric

c) Cessation of excessive sweating upon sleep

d) Frequency of episodes at least once per week

e) Positive family history

f) Impairment of daily activities

3) Hyperhidrosis Disease Severity Scale (HDSS) of 3 or 4

4) Sweat production of at least 50 mg over 5 minutes in each axilla assessed gravimetrically

5) Age ≥ 9 years

(2) Visit 2 (Randomization)

1) HDSS of 3 or 4

2) Sweat production of at least 50 mg over 5 minutes in each axilla assessed gravimetrically

3) Completed the Axillary Sweating Daily Diary (ASDD) or ASDD-C in patients aged ≤15 years at consent for Item 2 for at least 4 of the 7 days before randomization.

Exclusion Criteria

(1) Patients with a history and/or presence of the following diseases:

1) Hypersensitivity to glycopyrronium-containing products

2) Hypersensitivity to alcohol that requires drug treatment

(2) Patients with the following diseases:

1) Sjögren’s syndrome or Sicca syndrome

2) Glaucoma, inflammatory bowel disease, toxic megacolon, myasthenia gravis, or paralytic ileus

3) Ventricular arrhythmias, atrial fibrillation, atrial flutter. History of other supraventricular tachycardia with a ventricular rate greater than 100 (other than sinus tachycardia)

4) Urinary retention requiring catheterization due to prostatic hypertrophy or severe obstructive symptoms of prostatic hypertrophy

5) Any clinically significant cardiac, hepatic, renal, lung or blood disease

(3) Patients who have undergone the following therapies:

1) Any surgical treatment that influences the axillary perspiration

2) Nonprescription or prescription antiperspirants (including quasi-drugs containing aluminum chloride) for axilla within 14 days of Visit 1

3) Axillary iontophoresis within 28 days of Visit 1

4) Any surgical treatment or psychotherapy for hyperhidrosis

5) Botulinum toxin within 1 year of Visit 1

6) Cholinergics, anticholinergics, clonidine hydrochloride, mexiletine, or Chinese herbal drugs for the treatment of hyperhidrosis within 28 days of Visit 1

(4) Patients who have used any investigational medicinal product and/or participated in any clinical trial within 120 days before Visit 2

(5) Women who are pregnant, suspected of being pregnant, or lactating, patients who are not willing to use contraception during the study

(6) Patients who have used the investigational drug (including vehicle)

(7) Patients who have discontinued the study of GT

(8) Patients who are judged by the investigator to be ineligible as patients for any other reasons

**Table S1** Summary of treatment-related TEAE (safety population)

| TEAE^†^ | 3.75% GT  (n = 161) | 2.5% GT  (n = 168) | Vehicle  (n = 165) |
| --- | --- | --- | --- |
| Treatment-related TEAE, no. of patients (%) |  |  |  |
| All | 39 (24.2) | 26 (15.5) | 22 (13.3) |
| Death | 0 | 0 | 0 |
| Serious adverse event | 0 | 0 | 0 |
| Treatment modification  Discontinuation | 3 (1.9) | 0 | 0 |
| Interruption | 3 (1.9) | 2 (1.2) | 0 |
| Reduction | 0 | 0 | 0 |
| TEAE by intensity |  |  |  |
| Mild | 39 (24.2) | 25 (14.9) | 22 (13.3) |
| Moderate | 0 | 1 (0.6) | 0 |
| Severe | 0 | 0 | 0 |
| TEAE of special interest |  |  |  |
| Mydriasis/ blurred vision^‡^ | 18 (11.2) | 13 (7.7) | 4 (2.4) |
| Dysuria/urinary retention^§^ | 14 (8.7) | 8 (4.8) | 9 (5.5) |
| TEAE reported by >2% of patients in either group | |  |  |
| Mydriasis | 6 (3.7) | 6 (3.6) | 1 (0.6) |
| Photophobia | 10 (6.2) | 4 (2.4) | 1 (0.6) |
| Blurred vision | 2 (1.2) | 3 (1.8) | 2 (1.2) |
| Thirst | 8 (5.0) | 3 (1.8) | 5 (3.0) |
| Nasopharyngitis | 0 | 0 | 0 |
| Dysuria | 6 (3.7) | 5 (3.0) | 3 (1.8) |
| Pollakiuria | 5 (3.1) | 2 (1.2) | 5 (3.0) |
| Oropharyngeal pain | 1 (0.6) | 0 | 0 |
| TEAE occurring at application site | 3 (1.9) | 1 (0.6) | 2 (1.2) |
| Application site dermatitis | 1 (0.6) | 1 (0.6) | 0 |
| Application site irritation | 0 | 0 | 0 |
| Application site pruritus | 0 | 0 | 1 (0.6) |
| Folliculitis | 1 (0.6) | 0 | 0 |
| Application site folliculitis | 0 | 0 | 1 (0.6) |
| Wound | 0 | 0 | 0 |
| Acne | 0 | 0 | 0 |
| Eczema asteatotic | 1 (0.6) | 0 | 0 |

Data reported as n (%). ^†^Classified using MedDRA/J version 22.0. ^‡^Mydriasis, pupils unequal, hypermetropia, Blurred vision, and photophobia. ^§^Urinary hesitation, urinary retention, urine flow decreased, pollakiuria, dysuria, nocturia, and urine output decreased. GT, glycopyrronium tosylate; MedDRA, Medical Dictionary for Regulatory Activities; TEAE, treatment-emergent adverse event.
